# Evaluation of parametric models by the prediction error in colorectal cancer survival analysis 

**Published:** 2015

**Authors:** Ahmad Reza Baghestani, Mahmood Reza Gohari, Arezoo Orooji, Mohamad Amin Pourhoseingholi, Mohammad Reza Zali

**Affiliations:** 1 *Department of Biostatistics, Faculty of Paramedical Sciences, Shahid Beheshti University of Medical Sciences, Tehran, Iran.*; 2 *Department of Biostatistics, Iran University of Medical Sciences, Tehran, Iran.*; 3 *Gastroenterology and Liver Diseases Research Center, Research Institute for Gastroenterology and Liver Diseases, Shahid Beheshti University of Medical Sciences, Tehran, Iran*

**Keywords:** Parametric model, Prediction error, Apparent loss, Colorectal cancer

## Abstract

**Aim:**

The aim of this study is to determine the factors influencing predicted survival time for patients with colorectal cancer (CRC) using parametric models and select the best model by predicting error’s technique.

**Background:**

Survival models are statistical techniques to estimate or predict the overall time up to specific events. Prediction is important in medical science and the accuracy of prediction is determined by a measurement, generally based on loss functions, called prediction error.

**Patients and methods:**

A total of 600 colorectal cancer patients who admitted to the Cancer Registry Center of Gastroenterology and Liver Disease Research Center, Taleghani Hospital, Tehran, were followed at least for 5 years and have completed selected information for this study. Body Mass Index (BMI), Sex, family history of CRC, tumor site, stage of disease and histology of tumor included in the analysis. The survival time was compared by the Log-rank test and multivariate analysis was carried out using parametric models including Log normal, Weibull and Log logistic regression. For selecting the best model, the prediction error by apparent loss was used.

**Results:**

Log rank test showed a better survival for females, BMI more than 25, patients with early stage at diagnosis and patients with colon tumor site. Prediction error by apparent loss was estimated and indicated that Weibull model was the best one for multivariate analysis. BMI and Stage were independent prognostic factors, according to Weibull model.

**Conclusion:**

In this study, according to prediction error Weibull regression showed a better fit. Prediction error would be a criterion to select the best model with the ability to make predictions of prognostic factors in survival analysis.

## Introduction

 Colorectal cancer is the third most common cancer and cause of cancer death worldwide (1). The incidence and mortality of colorectal cancer are rising rapidly in Asian countries (2-4). Gastrointestinal cancers are the most important causes of mortality in Iran, which is located in the Middle East, Asia, and the burden of these cancers are increasing (5, 6) and CRC is not an exception (7). 

The prognosis of CRC is relatively good in terms of survival. Over the past 60 years, numerous claims have been made of variables being related to survival of colorectal cancer. Several studies have also considered independent prognostic factors, including age at diagnosis (8), sex (9), stage (10), histological grade (8) etc. 

Survival models or failure time models are statistical techniques to estimate the overall time up to specific events and find the related factors or predict the outcome. Prediction is important in medical science, because doctors need to estimate the survival of patients to choose the best treatment and it helps one to know about the disease condition in the future (11). The accuracy of prediction is determined by a measurement, generally based on loss functions, called prediction error. Recently, this technique developed to estimate the prediction error in survival analysis, in order to find the best model for analyzing the prognosis factors (12, 13). 

The aim of this study is to determine the factors influencing predicted survival time for patients with colorectal cancer using parametric models and select the best model by predicting error’s technique. 

## Patients and Methods

The data belongs to registered patients with colorectal carcinoma who admitted to the Cancer Registry Center of Gastroenterology and Liver Disease Research Center, Taleghani Hospital, Shahid Beheshti University of Medical Sciences; Tehran, Iran, in the period between 2002 to 2007. All patients were followed until January 1, 2007(as failure time) from their diagnosis by telephone contact (14, 15). Each patient was informed by a consent form for documenting his/her information in the Cancer Registry Center. The data of 600 patients who were followed at least for 5 years and have completed information selected for this study. Body Mass Index (BMI), Sex, family history of CRC, tumor site (colon, rectum), stage of disease (early, advanced) and histology of tumor (Mucinous, others) included in the analysis. The survival time was compared by the Log-rank test and multivariate analysis was carried out using parametric models including Log normal, Weibull and Log logistic regression. For selecting the best model, the prediction error by the apparent loss (12) was used in which smaller error indicates a better model. P<0.05 was considered as statistically significant and all analysis carried out using R software (16). 

**Table 1:** The result of log rank test for Univariate analysis

Prognostic Factors	Number of death	Number of patients	Mean (SE)	Test statistic	P-value
BMI					
Less than 25 More than 25	113(31.2) 38(16)	362(60.3) 238(39.7)	71.565(5.055) 128.032(9.719)	28.55	<0.0001
Tumor site					
Colon Rectum	96(23) 55(30.2)	418(69.7) 182(30.3)	109.166(6.99) 77.32(6.83)	4.936	0.026
Family history					
Yes No	88(25) 63(24.7)	345(57.5) 255(42.5)	74.557(6.523) 115.178(6.812)	2.034	0.154
sex					
Male Female	57(22.3) 94(27.3)	256(42.7) 344(57.3)	111.503(9.348) 88.114(8.334)	3.87	0.049
Stage					
Early Advance	57(18.8) 94(31.6)	303(50.5) 297(49.5)	117.436(7.768) 68.118(4.938)	25.537	<0.0001
Histology					
Mucinous Others	18(28.1) 133(24.8)	64(10.7) 536(89.3)	105.113(14.006) 99.799(6.369)	0.766	0.381

## Results

Among 600 patients, 344 were men (57.3%) and 256 were women (42.7%). Among 151 patients who died, 62.3% were men. The mean of survival for patients was 105.08 months (95% CI: 950.5-115.1) and the median was 94.5 months (95% CI: 58.6-130.4). Log rank test showed a better survival for females, BMI more than 25, patients with early stage at diagnosis and patients with colon tumor site (Table 1). 

All factors included in parametric models (Log normal, Weibull and Log logistic censored regression) and prediction error by apparent loss was estimated for each model consequently, which resulted as the 1.46 for Log logistic, 1.49 for Log normal and 1.28 for Weibull. Therefore, Weibull model was the best model among these parametric models (Table 2) and revealed that BMI and stage of disease were independent prognostic factors of CRC survival. The relative risk of death for patients in the advanced stage of disease is 2.27 times more compared to patients is in the early first stage and patients with low BMI (less than 25) were at higher risk of death, compared to those with BMI more than 25. The other variables were not significant. 

**Table 2:** The results of Weibull Censored Regression for Multivariate analysis

	RR	SE	P-value
BMI	2.2	0.16	<0.001
Tumor site	1.17	0.14	0.2
Family history	1.12	0.14	0.4
Sex	1.13	0.14	0.3
Stage	1.96	0.14	<0.001
Histology	1.08	0.21	0.7

Relative Risk, 2.Colon is as the reference group, 3. Others were as the reference group.

## Discussion

In this study, BMI and stage of the disease were prognostic factors of CRC survival, according to parametric regression model and the apparent loss prediction error indicated that Weibull model was the best option among parametric models to analyze the survival of CRC patients who admitted in Taleghani hospital. 

However, in the Log rank test, sex was a significant factor, multivariate model showed no relation between sex and survival of CRC patients. A population study on about 165,000 CRC patients in Germany reported a better survival for women (17). 

According to the histology type of tumor, Log rank test and Weibull model showed no difference in survival. This result is consistent to some studies (18, 19).

The Log rank analysis revealed a better survival of colon cancer, compared to rectal’s. However, this result was not significant in multivariate analysis. Other studies reported a better survival for colon cancer (20, 21). People with *rectal cancer tend to* be older and may have other serious health issues. Therefore, it would be the reason of different survival. 

Family history of CRC was another risk factor in our analysis. Although individuals with a family history of colorectal cancer are diagnosed more often than the general population (22), the study suggests that survival from colorectal cancer may not be worse (23) and the result of this analysis in both univariate and multivariate confirmed that. 

BMI was a prognostic factor of CRC survival in both Log rank and Weibull analysis and the patients with higher BMI had a better survival. A similar study suggested that underweight and obese women with colon cancer were at increased risk of death (24). 

In multivariate and univariate analysis, the effect of the cancer’s stage was significant on survival time. A similar study indicated that patients whose cancer is in the early stage have a better survival time (25).

In this study, we used a parametric model to analyze the survival rate of patients with CRC and select the best-appropriated model using prediction error. Parametric models are more flexible than Cox semi parametric model (26-28). Besides, prediction error would be criteria to select the best model with the ability to make predictions of prognostic factors in survival analysis.

## References

[1] Jemal A, Bray F, Center MM, Ferlay J, Ward E, Forman D (2011). Global cancer statistics. CA Cancer J Clin.

[2] Moghimi-Dehkordi B, Safaee A (2012). An overview of colorectal cancer survival rates and prognosis in Asia. World J Gastrointest Oncol.

[3] Pourhoseingholi MA (2012). Increased burden of colorectal cancer in Asia. World J Gastrointest Oncol.

[4] Pourhoseingholi MA, Vahedi M, Baghestani AR (2015). Burden of gastrointestinal cancer in Asia; an overview. Gastroenterol Hepatol Bed Bench.

[5] Pourhoseingholi MA, Vahedi M, Moghimi-Dehkordi B, Pourhoseingholi A, Ghafarnejad F, Maserat E (2009). Burden of hospitalization for gastrointestinal tract cancer patients - Results from a cross-sectional study in Tehran. Asian Pac J Cancer Prev.

[6] Pourhoseingholi MA, Fazeli Z, Ashtari S, Bavand-Pour FS (2013). Mortality trends of gastrointestinal cancers in Iranian population. Gastroenterol Hepatol Bed Bench.

[7] Pourhoseingholi MA, Faghihzadeh S, Hajizadeh E, Abadi A, Zali MR (2009). Bayesian estimation of colorectal cancer mortality in the presence of misclassification in Iran. Asian Pac J Cancer Prev.

[8] Weiss JM, Pfau PR, O’Connor ES, King J, LoConte N, Kennedy G (2011). Mortality by stage for right- versus left-sided colon cancer: analysis of surveillance, epidemiology, and end results - Medicare data. J Clin Oncol.

[9] Coleman M, Quaresma M, Berrino F, Lutz JM, De Angelis R, Capocaccia R (2008). Cancer survival in five continents: a worldwide population-based study (CONCORD). Lancet Oncol.

[10] Nedrebø B, Søreide K, Eriksen M, Dørum L, Kvaløy J, Søreide J (2011). Survival effect of implementing national treatment strategies for curatively resected colonic and rectal cancer. J Surg.

[11] Henderson R, Keiding N (2005). Individual survival time prediction using statistical models. J Med Ethics.

[12] Gerds TA, Schumacher M (2007). Efron-type measures of prediction error for survival analysis. Biometrics.

[13] Lawless JF, Yuan Y (2010). Estimation of prediction error for survival models. Stat Med.

[14] Azadeh S, Moghimi-Dehkordi B, Fatem SR, Pourhoseingholi MA, Ghiasi S, Zali MR (2008). Colorectal cancer in Iran: an epidemiological study. Asian Pac J Cancer Prev.

[15] Moghimi-Dehkordi B, Safaee A, Zali MR (2008). Prognostic factors in 1,138 Iranian colorectal cancer patients. Int J Colorectal Dis.

[16] Hothorn T, Everitt BS (2014). A Handbook of Statistical Analyses Using R.

[17] Majek O, Gondos A, Jansen L, Emrich K, Holleczek B, Katalinic A (2013). Sex differences in colorectal cancer survival: population-based analysis of 164,996 colorectal cancer patients in Germany. PLoS One.

[18] Nilsson KR, Berenholtz SM, Dorman T, Garrett P, Kaufman HS, Pronovost PJ (2002). Preoperative predictors of blood transfusion in colorectal cancer surgery. J Gastrointest Surg.

[19] Liang H, Wang XN, Wang BG, Pan Y, Liu N, Wang DC (2006). Prognostic factors of young patients with colon cancer after surgery. World J Gastroenterol.

[20] Wray CM, Ziogas A, Hinojosa MW, LeH, Stamos MJ, Zell JA (2009). Tumor subsite location within the colon is prognostic for survival after colon cancer diagnosis. DisColon Rectum.

[21] Meguid RA, Slidell MB, Wolfgang CL, Ahuja N (2008). Is there a difference in survival between right-versus left-sided coloncancers?. Ann SurgOncol.

[22] Safaee A, Moghimi-Dehkordi B, Pourhoseingholi MA, Vahedi M, Maserat E, Ghiasi S (2010). Risk of colorectal cancer in relatives: A case control study. Indian J Cancer.

[23] Kirchhoff AC, Newcomb PA, Trentham-Dietz A, Nichols HB, Hampton JM (2008). Family history and colorectal cancer survival in women. Fam Cancer.

[24] Doria-Rose VP, Newcomb PA, Morimoto LM, Hampton JM, Trentham-Dietz A (2006). Body mass index and the risk of death following the diagnosis of colorectal cancer in postmenopausal women (United States). Cancer Causes Control.

[25] Maringe C, Walters S, Rachet B, Butler J, Fields T, Finan P (2013). Stage at diagnosis and colorectal cancer survival in six high-income countries: a population-based study of patients diagnosed during 2000-2007. Acta Oncol.

[26] Pourhoseingholi MA, Hajizadeh E, Moghimi Dehkordi B, Safaee A, Abadi A, Zali MR (2007). Comparing Cox regression and parametric models for survival of patients with gastric carcinoma. Asian Pac J Cancer Prev.

[27] Moghimi-Dehkordi B, Safaee A, Pourhoseingholi MA, Fatemi R, Tabeie Z, Zali MR (2008). Statistical comparison of survival models for analysis of cancer data. Asian Pac J Cancer Prev.

[28] Pourhoseingholi MA, Moghimi-Dehkordi B, Safaee A, Hajizadeh E, Solhpour A, Zali MR (2009). Progostic factors in gastric cancer using log-normal censored regression model. Indian J Med Res.

